# Adapting to Modernity. River Engineering and Emotional Engineering in 1950s Sweden

**DOI:** 10.1007/s00048-026-00443-x

**Published:** 2026-03-10

**Authors:** Fabian Zimmer

**Affiliations:** Fachgebiet Technikgeschichte, Technische Universtität Berlin, Straße des 17. Juni 135, 10623 Berlin, Deutschland

**Keywords:** Adaptation, Social engineering, History of emotions, Hydropower, Public relations, Technology acceptance, Anpassung, Social engineering, Emotionsgeschichte, Wasserkraft, Public Relations, Technikakzeptanz

## Abstract

In the mid-1950s, Vattenfall, the Swedish State Power Board, faced a severe acceptance crisis. In the postwar years, Vattenfall had become one of the main drivers of Sweden’s technological modernization through electrification and the exploitation of hydropower resources in Northern Sweden. Yet, this postwar hydro boom faced increasingly powerful protests from both civil society and local communities, putting Vattenfall’s construction plans at stake and spurring an intense public relations campaign. This paper examines Vattenfall’s PR materials, and especially the large body of films produced in the context of this acceptance crisis. The first half of the paper offers a close reading of these materials, demonstrating how Vattenfall addressed conflicts around hydropower exploitation as a matter of individual “adaptation” to modernity. With this framing, Vattenfall drew upon a much broader adaptation discourse, analyzed in the second half of the paper. This discourse shaped public and political debates about technological and social modernization in postwar Sweden and corresponded with the social engineering ideals of the “people’s home” (*folkhemmet*) and Sweden’s welfare state. Social scientists, politicians, managers, trade unionists, and others developed the idea that the challenges of modernization should be met by the adaptation of people’s individual behaviors and emotions to their rapidly changing social and technological environments. Arguing for a history of emotions approach in the study of histories of “technology acceptance,” this paper thus demonstrates how river engineering in postwar Sweden relied on emotional engineering techniques through PR: framing social conflicts over hydropower as individual adaptation processes facilitated the depoliticization of technological choice.

## Abstract (de)

Mitte der 1950er Jahre geriet der schwedische staatliche Energieversorger Vattenfall in eine schweren Akzeptanzkrise. Vattenfall war nach dem Zweiten Weltkrieg mit der Elektrifizierung des Landes und der Ausbeutung der Wasserkraftressourcen in Nordschweden zu einem der wichtigsten Treiber technischer Modernisierung in Schweden geworden. Dieser Wasserkraftboom stieß jedoch auf zunehmende Proteste der Zivilgesellschaft und der lokalen Bevölkerung. Sie gefährdeten ernsthaft Vattenfalls weitere Ausbaupläne und regten das Unternehmen zu intensiver Öffentlichkeitsarbeit an. In diesem Beitrag untersuche ich PR-Material von Vattenfall, insbesondere die zahlreichen Filme, die das Unternehmen im Zusammenhang mit dieser Akzeptanzkrise produzierte. Anhand dieser Materialien zeige ich zunächst, wie Vattenfall Konflikte um die Nutzung der Wasserkraft als eine Frage der individuellen „Anpassung“ an die Moderne darstellte. Mit diesem Framing stützte sich Vattenfall auf einen wesentlich breiteren Anpassungsdiskurs, den ich anschließend analysiere. Dieser Diskurs prägte die öffentlichen und politischen Debatten über die technische und soziale Modernisierung im Nachkriegsschweden und war Ausdruck von für den schwedischen Wohlfahrtsstaat charakteristische Ideale des *social engineering*. Sozialwissenschaftler, Politiker, Manager, Gewerkschafter und andere entwickelten die Idee, dass die Herausforderungen der Moderne bewältigt werden sollten, indem sich die Individuen in ihrem Verhalten und ihren Emotionen an die sich rasch verändernde soziale und technologische Umgebung anpassten. Dieser Beitrag plädiert damit für einen emotionshistorischen Ansatz in der Geschichte der „Technikakzeptanz“ und zeigt auf, wie sich Wasserbau im Nachkriegsschweden auf die emotionale Ingenieurskunst der Public Relations stützte: Indem gesellschaftliche Konflikte um den Ausbau der Wasserkraft als individuelle Anpassungsprozesse gerahmt wurden, sollten politische Entscheidungen über technische Entwicklung entpolitisiert werden.

## Fear, Technology Acceptance, and the History of Emotions

In 1955, *Vi i Vattenfall *(literally: We in Vattenfall), published an open “Letter to a newly employed ‘lake regulator.’” The letter in the internal staff magazine for employees and workers at the Swedish State Power Board, better known as Vattenfall, was authored by Jonas Norrby, at the time head of Vattenfall’s “lake regulation department” (*regleringsavdelning*), which was responsible for land surveying, land acquisition, resettlement, and similar preparatory measures for the construction of new dams and other hydraulic infrastructure destined to turn natural lakes and river stretches into water power reservoirs. In his letter, Norrby painted a dramatic image of the public opinion facing Vattenfall’s “lake regulators” in the 1950s:

“The first time you face the criticism, you may be shocked. Your intentions have been the best, your will exemplary. … And then one morning you might hear that there should be a bounty on your head and that of your comrades. That you are an invader, one who comes to cart people away from home and hearth simply because the common good demands it. … You think of yourself as someone who serves the common good, and understandably enough you are discouraged when others don’t understand things the way you do. You think the arguments are so clear. You lose your desire for this job, you are a technician, you want to be a technician. ‘Rational and economical’ is your guiding star.” (Norrby 1955)^1^

A similar experience from the same year is reported by Ivan Christofferson, who worked in Vattenfall’s PR department and later recalled how he was threatened by local people during a shoot for a documentary short film about lake regulations:

“Here and there, men with axes on their backs came out of the forest, surrounded us and interrogated us in a threatening tone about what we were going to do and who we were. Were we Vattenfall people [*vattenfallare*] perhaps? The fists were up. The film director truthfully answered no. So did the photographer. I also said no. I lied because I was scared to death. Then the villagers started talking about how Vattenfall had been there and ‘bought up the farms. At low prices.’ A man from the Claims Settlement Department [*Skaderegleringsavdelningen*] had said that if they didn’t agree to those terms, there would be compulsory acquisition and even worse payment. All three of us were badly affected. … I was hugely ashamed of being in Vattenfall [*vara vattenfallare*] for a long time afterwards.” (Forsgren 1993: 66)

Also writing in hindsight, Charlie Cederholm, head of Vattenfall’s PR department at the time, confessed that the conflicts of the 1950s regularly put Vattenfall’s management and the PR service in a state of “despair” (*misströstan*) and “powerlessness” (*vanmakt*).^2^

These quotes let us catch a glimpse of the emotional impact of a severe acceptance crisis which struck Vattenfall during the 1950s. This crisis developed at the conjuncture of technical, economic, and political trends. Vattenfall had been a central actor in the exploitation of Swedish hydropower resources already since its foundation as a state agency in 1909, but after World War II, it became the major driving force in a rapid expansion of the electric grid and hydraulic infrastructure in Sweden, with most construction works taking place in remote Northern Sweden. However, alongside this boom in hydropower constructions, Vattenfall faced increasing public protest. During the 1950s, several new laws strengthened the position of nature conservation and heritage organizations as well as opportunities for the participation of local communities. And Swedish civil society was ready to take advantage of these opportunities. Most notably, in 1956 an entire “lake regulation” project was toppled after the protest of a coalition of local initiatives and regional and national conservation and heritage organizations and institutions. Due to public pressure, and in order to assure future planning, Vattenfall even saw itself forced to negotiate a sort of “peace treaty” in 1962 with various groups opposing the exploitation of hydropower—the so-called “Peace of Sarek” (*Freden i Sarek*).^3^

Despair, powerlessness, fear, shame, and discouragement on the part of Vattenfall’s engineers, management, and PR officials on the one hand, mistrust and anger on the part of local people facing resettlement on the other hand—the emotions expressed in the above quotes are indeed remarkable, perhaps even surprising, as they run counter to conventional narratives of “technology acceptance.” Such narratives tend to portray engineers, managers, and other proponents of technology as rational and controlled decision-makers, while the public is oftentimes imagined as irrational, uninformed, passive, and driven by fear. The “common cliché of anxious enemies of technology” has deeply informed the way we imagine the emotions of groups protesting against new technologies (Heßler & Hitzer 2019: 185; cf. Rieger 2003). In reversing this commonplace attribution of fear, the above quotes from the 1950s alert us to the emotional attentiveness required from historians dealing with “technology acceptance.” They give us aa sense of how debates about technological change take place in a much more varied emotional landscape than the clichéd image of the rational engineer and the fearful public would suggest.^4^ This compels historians of technology to engage with approaches from the history of emotions—in a dialogue which is only gradually emerging (cf. Heßler 2020).

In this paper, I contribute to this dialogue, by closely analyzing and contextualizing how Vattenfall addressed the acceptance crisis of the 1950s through public relations, and especially through the medium of film. On the following pages, I will argue that Vattenfall used films as a social engineering tool, or more precisely as an “emotional engineering technique,” as Anja Laukötter (2016) has put it. They were designed to shape and regulate the emotions of the public concerning the construction of hydraulic infrastructure, in order to gain and maintain acceptance for Vattenfall’s construction plans. In keeping with approaches from the history of emotions, I do not aim to reconstruct in any detail the actual emotions of Vattenfall’s employees or those of their adversaries. Rather, what I analyze here are “systems of feeling” shared by specific “emotional communities,” as medievalist Barbara Rosenwein has called them in her pioneering work on the history of emotions. In analyzing and contextualizing films, I aim

“to uncover systems of feeling: what these communities (and the individuals within them) define and assess as valuable or harmful to them; the evaluations that they make about others’ emotions; the nature of the affective bonds between people that they recognize; and the modes of emotional expression that they expect, encourage, tolerate, and deplore.” (Rosenwein 2002: 842)

I interpret Vattenfall’s PR and film work as parts of a specific “emotional practice” (Scheer 2012), through which the company sought to align public appraisals of hydropower constructions with a specific “system of feeling.” I ask: How did Vattenfall conceptualize and define emotions? And how did the company put these conceptualizations into practice as a set of emotional rules and emotional role models, mediated through film as an “emotional engineering technique”?

So, what I trace here, in a sense, is Vattenfall’s take on matters of “technology acceptance” and how Vattenfall tried to foster this acceptance through public communication. Vattenfall only very rarely labelled its PR activities as attempts to foster “acceptance.” Rather, the Agency framed them as guidance that would help individuals and the public in “adapting” to a modern age powered by hydroelectricity. The first half of this paper is dedicated to carving out this adaptation discourse in a close reading of Vattenfall’s PR films. Then, in the second half of the paper, I will demonstrate that by framing conflicts over technological choice in terms of “adaptation,” Vattenfall took advantage of a broad discourse on “adaptation” in postwar Swedish industry, sociology, and politics, which was closely linked to the wider cultural context and the ideals of the postwar Swedish welfare state. It is against this backdrop, I argue, that the adaptation discourse deployed by Vattenfall to foster acceptance of its hydroelectricity program could expect tremendous plausibility.

## Industrial Films as an Emotional Engineering Technique

At the end of the 1950s, Vattenfall possessed the largest PR department among Sweden’s businesses. To a contemporary observer the exceptional size of Vattenfall’s “press service” (*presstjänst*) was easily explained “with regard to the particularly intricate public opinion issues that this organization has to deal with in connection with water construction and water regulation” (Kjellström 1959: 45). However, neither the importance of Vattenfall’s press service, nor that it was largely concerned with “public opinion issues” was self-evident from the outset. This was a development that only occurred over the course of the 1950s—and indeed in close conjunction with the growing resistance and public protest Vattenfall faced in this period. When Vattenfall’s press service was first founded in 1948, it consisted of just one person (as opposed to the eleven people employed by 1959), and its work was focused on internal affairs.^5^ Its integration into the State Power Board’s “administrative office” (*kanslibyrån*)—which was responsible for human resources and other internal affairs—already speaks to this. Over the course of the 1950s, however, the press service took over an increasing array of duties and especially the production and distribution of films came to play a vital role in its PR activities. In a 1959 document, Charlie Cederholm, the head of the press service, even defined it as “an organ for the company’s external PR-work.” He therefore deemed it “natural that film has gradually become an important means of contact with the public.” The press service had taken over the centralized production and distribution of industrial films in 1954 and had immediately geared them to purposes of external communication. Since the mid-1950s, all films produced or commissioned by Vattenfall—“even a purely technical one”—were to be produced in collaboration with renowned film companies for a broad public.^6^ The result of this new approach was over twenty high-quality short documentaries released between 1954 and 1959, which circulated in the pre-program of national cinemas, toured through the countryside and various local divisions of the company, and were lent on 16 mm to various organizations, associations, and companies—amounting to a total audience of about 3.1 million people in just one year (1959), according to the press service.^7^ Vattenfall is thus “the real standout in Swedish industrial film production in the 1950s,” as film and media scholar Mats Björkin has recently put it (2022: 85).

While the films produced by Vattenfall in this period treated a variety of topics, including regional electricity distribution or specific prestige construction projects, the issues and conflicts around lake regulations in Northern Sweden were *the* central topic in Vattenfall’s films from the 1950s, and were addressed in no fewer than ten films.^8^ In the following analysis, I take a closer look at three of these films in the chronological order of their production and analyze how Vattenfall gradually refined its narrative in an attempt to shape the emotions of the public: from first acknowledging that the hydroelectric modernization of Northern Sweden could cause the affected individuals problems—framed as emotional adaptation problems—in *Öden bortom horisonten* (Destinies beyond the horizon, premiered 1956), to suggesting a rational and trustful approach to these emotional problems in *Den nya sjön *(The new lake, 1957), to eventually staging emotional role models for adaptation in a fictional plot in *Det nya ansiktet* (The new face, 1959).

### Acknowledging Adaptation Problems—*Öden bortom horisonten*

Written and directed by the renowned short film director Gösta Werner and first screened in cinemas in 1956, *Öden bortom horisonten* was among the first films commissioned after Vattenfall’s press service had centralized and reorganized film production.^9^ Conceived as a “general good-will-film … which could help educate the public [*upplysa folk*] about the lake regulations and what we are doing to help the people affected,”^10^ it approached the most pressing issue for Vattenfall’s public reputation at the time. And the way it did so constituted a clear break with earlier representations of hydropower exploitation in Northern Sweden.

*Öden bortom horisonten* is a film about modernization. It situates the issue of lake regulations within a sweeping modernization narrative about the Swedish North: in the first few minutes, the beautiful color cinematography and the narrator paint a pastoral image of Northern Sweden:

“unknown, remote, romantic. Humming with green forests, roaring with white waterfalls. Man came early to the mountain regions of Norrland. In the wake of the retreating ice caps came the flocks of primitive hunters who roamed freely across the plains, hunting elk and reindeer. Perhaps the Lapps are their descendants. The Lapps, one of the world’s last free-roaming peoples, for a long time the unchallenged masters of Norrland.”

But soon enough the intrusion of modernity interrupts this seemingly static state: “But the new times came, with railways through Lapland’s roadless expanses. … The new times excavated iron ore deep down in the rock. The new times built ironworks at the end of the railway, and sawmills and pulp mills all along the coast of Norrland.” Up until this point, the film thus uses a very conventional image of the modernization of Northern Sweden just as the romantic and industrial colonialist imagination would have wished it to be: as the “land of the future” (*framtidslandet*), where “untouched” nature and “primitive” culture clashed with economic promise and fortune (cf. Sörlin 1988; Öhman 2007).

**Fig. 1:**
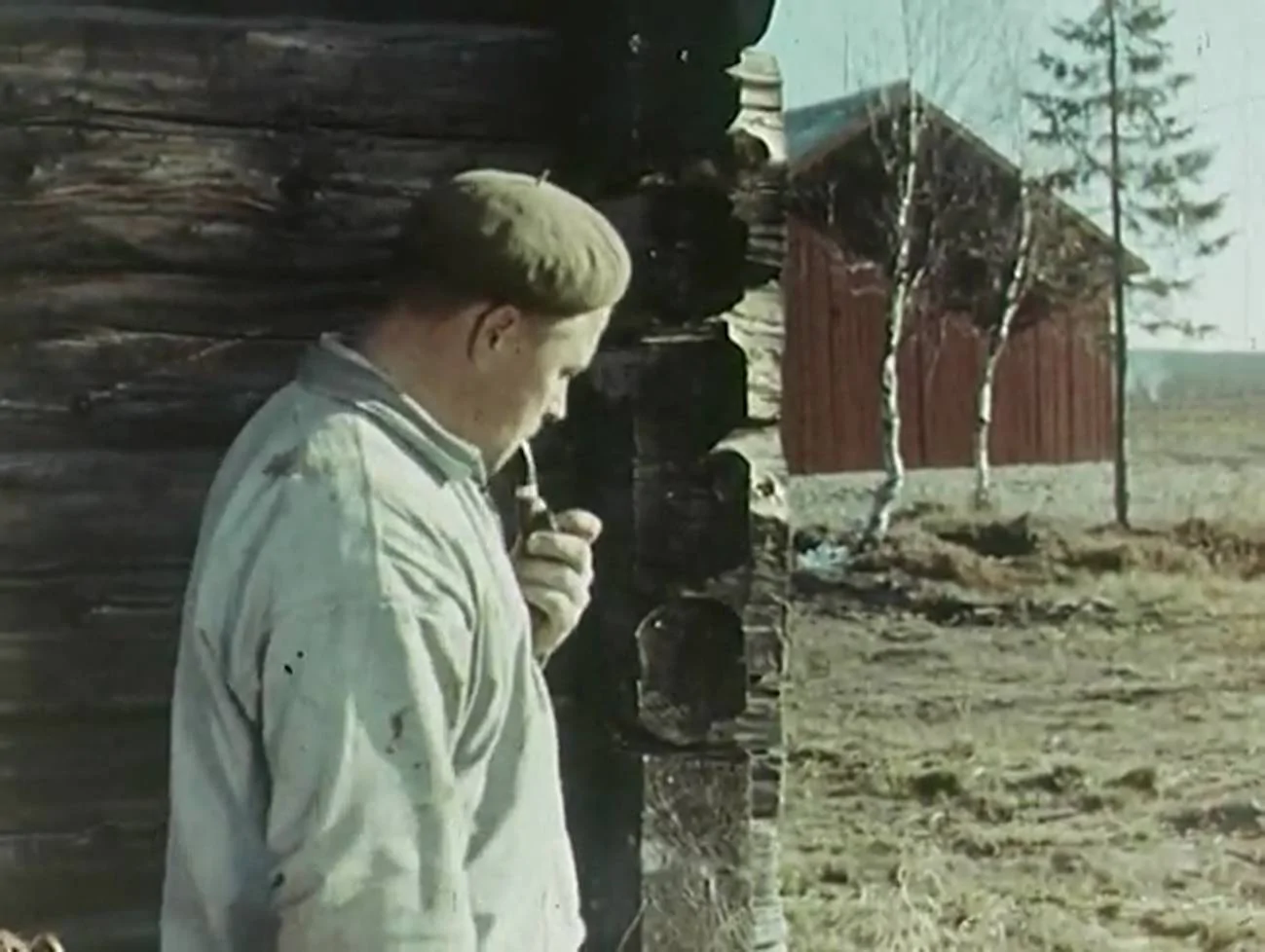
The farmer reflecting on his new life after resettlement in *Öden bortom horisonten*

It is only when the film then turns to the issue of lake regulations and resettlement in Northern Sweden that this modernization of the “new times” (*den nya tiden*) becomes problematic. “[I]t is here one encounters a significant, up here clear-cut problem: the mountain dweller [*fjällbon*], the human factor in the struggle for the kilowatts.” The film then portrays the destinies of these “mountain dwellers” along the lakes of Northern Sweden—by which the film does not mean the Sami (the “Lapps” in the above quote), but the ethnically Swedish inhabitants of the North. It portrays the destinies of those who remain but whose livelihood on the lake is profoundly altered, or of those who face resettlement in the wake of a lake regulation. The “human factor” is most poignantly portrayed in the sequence which leads to the conclusion of the film. Here, the camera closes in on an anonymous farmer who has recently resettled to Nyängen, a model village in which Vattenfall offered farmers resettlement to land on a reclaimed bog on the outskirts of the village of Sorsele (cf. *Vi i Vattenfall* 1953; see also below). As we see the farmer outside his new barn, pondering and puffing on his pipe (see Fig. 1), the narrator asks: “He is now better off economically, but how about his well-being [*trivseln*]?—And his wife? Is she doing better or worse?” The film refrains from giving an answer to these questions: “No one can answer this either. She has accepted resettlement and change and what it entails. They both have sacrificed something, something that once was essential for them, and without it they now start over again.”

This modernization narrative in *Öden bortom horisonten* is unprecedented in Vattenfall’s official narratives. Earlier films and other media representations did not address resettlement at all, to say nothing of its emotional implications for those uprooted. Rather, they had portrayed technological modernization as a heroic national feat and as an immaculate promise, in which the question of individual “well-being” (*trivseln*) simply did not arise. In short, earlier narratives of hydropower in Sweden depicted modernization in the perspective of the “technological sublime,” as historian David Nye (1994) has called it (cf. Fridlund 1998; Zimmer 2019, 2024). Now, by contrast, with *Öden bortom horisonten* and later films, Vattenfall explicitly and publicly acknowledged that lake regulations not only served the common good but also indeed caused problems. These problems called for resolution. However, it was not technical or political solutions that Vattenfall’s films would highlight, but rather individual behavioral and emotional adaptation to the challenges of modernization.

Even while zooming close up on individual characters, *Öden bortom horisonten* maintains a clear division between the film characters and the audience. The audience targeted by the film was clearly a metropolitan, urban one—as the film compares the encroachments brought by lake regulations in the Swedish North with the simultaneous urban development project in central Stockholm, the “regulation” of Norrmalm (*norrmalmsregleringen*). Adaptation to and acceptance of the “new times” is cast as an issue which ultimately concerns the “mountain dweller” of the Swedish North. Already in the exposition, when introducing the film’s sweeping modernization story, the narrator characterizes the inhabitants of Northern Sweden as “individualists,” people who “had difficulty adapting, subordinating themselves.” In the above quoted sequence then, adaptation has already happened: for want of an alternative the farmer’s wife has “accepted resettlement and change.” Acceptance is depicted as a fatalist submission to the eponymous “Destinies beyond the horizon”—a fate which cannot be altered. *Öden bortom horisonten* is therefore a film *about *adaptation, rather than a film* for *adaptation—quite opposed to the two subsequent films on lake regulations, to which I now turn.

### Adaptation Through Rationalization—*Den nya sjön*

*Den nya sjön* (The new lake), produced between 1956 and 1957 and screened in the pre-program of national cinemas in 1959,^11^ was Vattenfall’s next filmic foray into the issues of lake regulations. This film follows a similar modernization narrative to *Öden bortom horisonten*, as is already announced by its title. And likewise, the acknowledgement of the problematic character of lake regulations is integral to its plot. Already in its exposition, *Den nya sjön *addresses the emotional impact provoked by lake regulations. The film narrates how news of a planned lake regulation arrives in a village on the shores of the respective lake; and it vividly portrays the reactions of three villagers, who enter a debate on the prospective water level: “One started to guess hither and thither: ‘Here will be the water.’ ‘No, that high!’ ‘No, even higher! It can be as high as the roof of your house there!’” This scene is followed by a close-up portrait of one of the villagers gazing into an uncertain future while the narrator expresses understanding for his feelings: “It was no wonder that people got a little anxious [*orolig*].”

Following the “common cliché of anxious enemies of technology” (Heßler & Hitzer 2019: 185), the film thus portrays the emotional reaction of the affected people as characterized by sorrow, anxiety, and fear—and not for instance by mistrust or anger, which were also on the spectrum of emotions faced by Vattenfall’s “lake regulators” out in the field, as I demonstrated in the introduction. This framing comes with certain narrative advantages. For one, it places Vattenfall in the generous position of supporting and offering help, as opposed to the defensive and inferior position in which the Agency found itself when faced with the public protest of the period. And secondly, this framing makes it possible for the film to present role models for how to overcome the painful emotions produced by lake regulations.

**Fig. 2:**
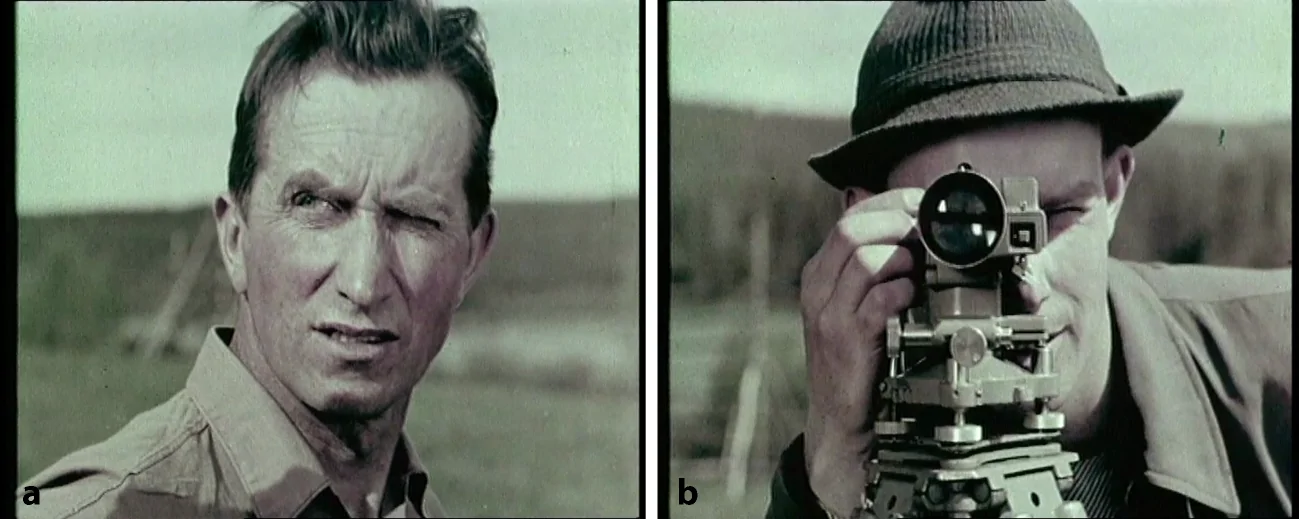
The anxious gaze of the villager and the measuring gaze of Vattenfall’s land surveyor *in Den nya sjön*

**Fig. 3:**
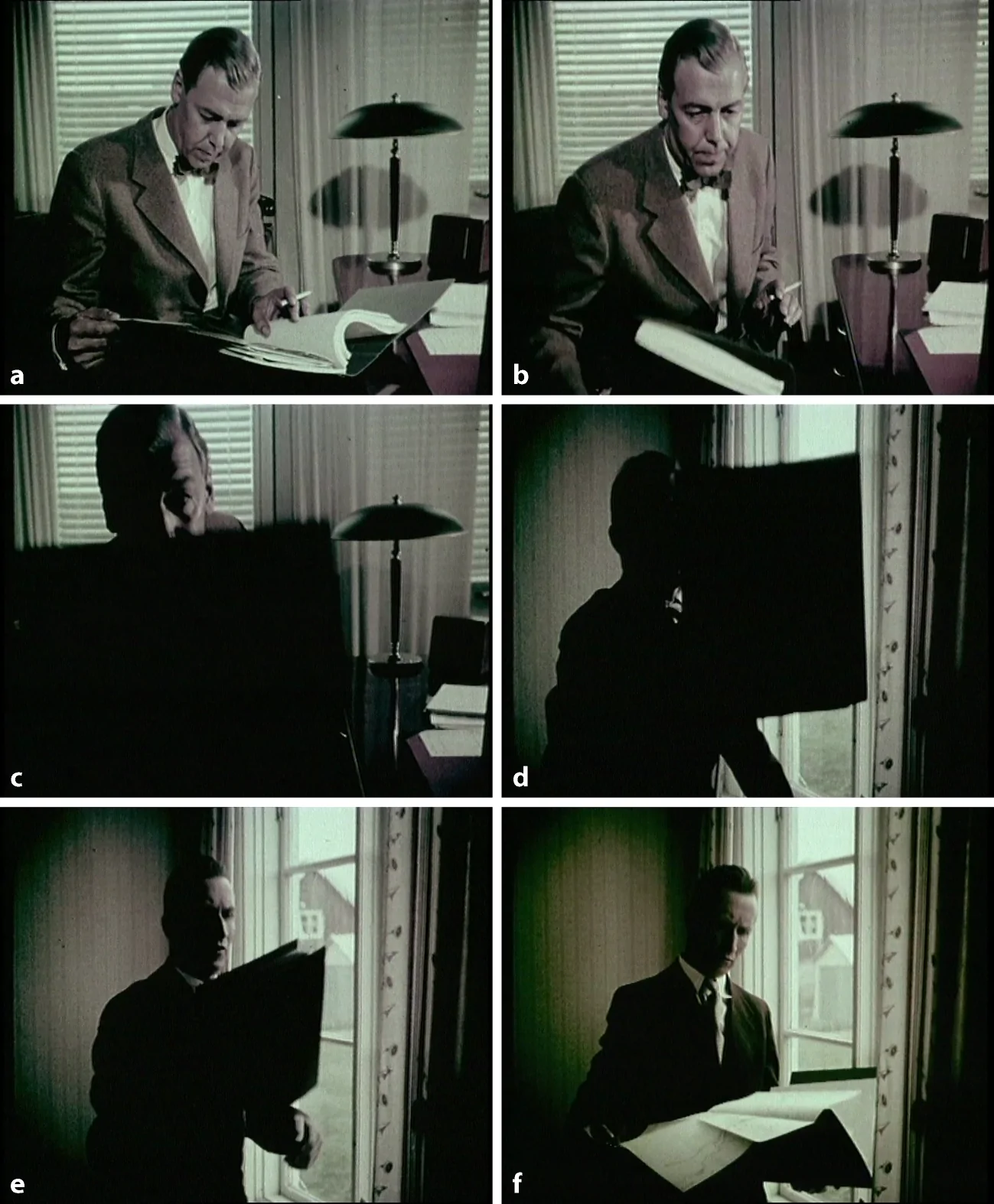
The land survey documentation folder as a token of reliability in *Den nya sjön*

The film suggests that a successful adaptation to the “new times” is possible through a process of rationalization and the building of mutual trust. It introduces this rationalization process with a striking match cut (see Fig. 2): the anxious gaze of the villager in the opening scenes is juxtaposed with the measuring eye of one of Vattenfall’s land surveyors. This frame introduces a long sequence portraying the detailed surveying and documentation process in which a living landscape is objectified into words, numbers, and maps, and eventually filed conveniently, in dozens of folders. Another match cut concludes this sequence: one of the folders is carefully studied by the aforementioned head of Vattenfall’s “regulation department,” Jonas Norrby, who then puts the folder back on its shelf, so covering up the camera’s lens. The folder is then taken off the shelf again—this time, in the affected village, where another copy is held (see Fig. 3). The folders and the knowledge conscientiously assembled in them thus become a token of reliability and transparency and form the basis for the passage from an emotional appraisal of the situation to a rational assessment. The narrator concludes the sequence by saying: “Now, there is no need to guess anymore. On the ground, sticks show where the new shoreline will go.”

However, in this sequence the film takes great care to depict the land surveyors not as agents of a cold and inhumane rationalization, but rather as approachable and trustworthy people. Testifying to their harmlessness, the camera shows the surveyors interviewing the village’s children and sitting down for a round of coffee together with some villagers—sharing a *fika*, the epitome of Swedish sociability. And conversely, the narrator highlights that “the village elder”—the anxious man shown in Fig. 2—“willingly answered all questions concerning the common interests of the village,” thus demonstrating the same rational and self-controlled behavior as Vattenfall’s land surveyors.

That Vattenfall is to be seen as a reliable, transparent, trustworthy, and approachable partner in issues related to lake regulations is also the message conveyed in the second half of the film, which portrays Vattenfall’s “local office” (*lokalkontor*) at another lake that has already been “regulated.” In elaborate before-and-after comparisons, the film presents various individual “solutions” that have been found for problems with water supply, timber floating, fishing, laundry facilities, and resettlement. The local office is thus staged as a support for individual adaptation processes, which furnishes appropriate technological or monetary solutions. Still, people need to adapt to the solutions presented by the local office. For instance, the film presents a newly constructed bathing area while the narrator slips into a more general theory of adaptation: “Some of the children may miss the old bathing areas. But they will soon forget this when they get the new ones. It’s easy for the young ones to adapt.”

As much as the harmonious image conjured in *Den nya sjön* contrasts with the mistrust and anger Vattenfall faced during the period, it was quite in line with Vattenfall’s PR efforts in other media. For instance, the self-control exerted by the “lake regulators” in the film was no mere fiction, but was substantiated by calls for exactly this type of behavior in Vattenfall’s internal communications. In the “Letter to a newly employed ‘lake regulator’” in Vattenfall’s staff magazine *Vi i Vattenfall* (which I quoted in the introduction), Jonas Norrby (1955) urged his employees to bear in mind the “great responsibility towards the people and interests affected by our work” and offered a list of “guidelines,” which amounted to the very same virtues of transparency and trustworthiness conveyed as the central message in *Den nya sjön.* Likewise, a reportage about the lake regulation department which followed in the same issue of *Vi i Vattenfall* ascribed a similarly controlled behavior to the local population, claiming that “among ‘lake regulators’ there is great admiration for the objectivity and calmness with which the community’s own spokesmen speak for their cause” (*Vi i Vattenfall* 1955a: 21).

Trustworthiness and transparency were just as central in Vattenfall’s external communications, besides film screenings. Not only the behavior of the Agency’s “lake regulators” in their contact with local people, but also for instance Vattenfall’s tourism activities were geared towards this goal. Starting in 1956, Vattenfall opened an increasing number of its power plants for public visits and employed young women as “waterfall hostesses” (*kraftverksvärdinnor/vattenfallsvärdinnor*), to replace the (all-male) technical and operational staff that had taken care of occasional tourists so far. The choice of female “hostesses” constituted a deliberate attempt to make tourist visits more “pleasant and agreeable” (*Vi i Vattenfall* 1956b; cf. Zimmer 2021). Furthermore, Vattenfall made an effort to cultivate an image of its sustained support for individuals affected by resettlement. The farmer at Nyängen, portrayed in *Öden bortom horisonten*, “Valter Brännlund, a farmer in his prime,” was closely followed by Vattenfall’s press service (cf. 1953, 1955b; as well as in the internal newsreel *Det hände 1955*). Clearly, the project at Nyängen was a matter of prestige and of demonstrating generosity. The sixteen allotments originally planned “to help a larger group of people who have their properties completely destroyed” (*Vi i Vattenfall* 1955a: 22) apparently never materialized and the “village” never expanded beyond the three households that moved there in 1955.

### Role Models for Adaptation—*Det nya ansiktet*

The final film on lake regulations produced by Vattenfall, *Det nya ansiktet*, had been considered by Vattenfall’s press service since at least 1956, but work on it did not pick up pace until 1958 and it was screened as a pre-program film the following year.^12^*Det nya ansiktet* addressed a different issue in relation to “lake regulations” and accordingly also aimed for a different audience. While *Öden bortom horisonten* was apparently aimed at a metropolitan audience with sympathies for the problems of Northern Swedish farmers, and while *Den nya sjön* may additionally have been aimed at local people who could identify with the stories told of resettlement, *Det nya ansiktet* had originally been projected under the working title *Naturskydd *(Nature conservation).^13^ It thus aimed to convince an audience sympathizing with the ideals and the institutions of nature conservation, which constituted the most serious threat to Vattenfall’s hydro expansion plans during the 1950s. Accordingly, its focus is not resettlement in the wake of lake regulations, but the latter’s impact on nature and landscape.

**Fig. 4:**
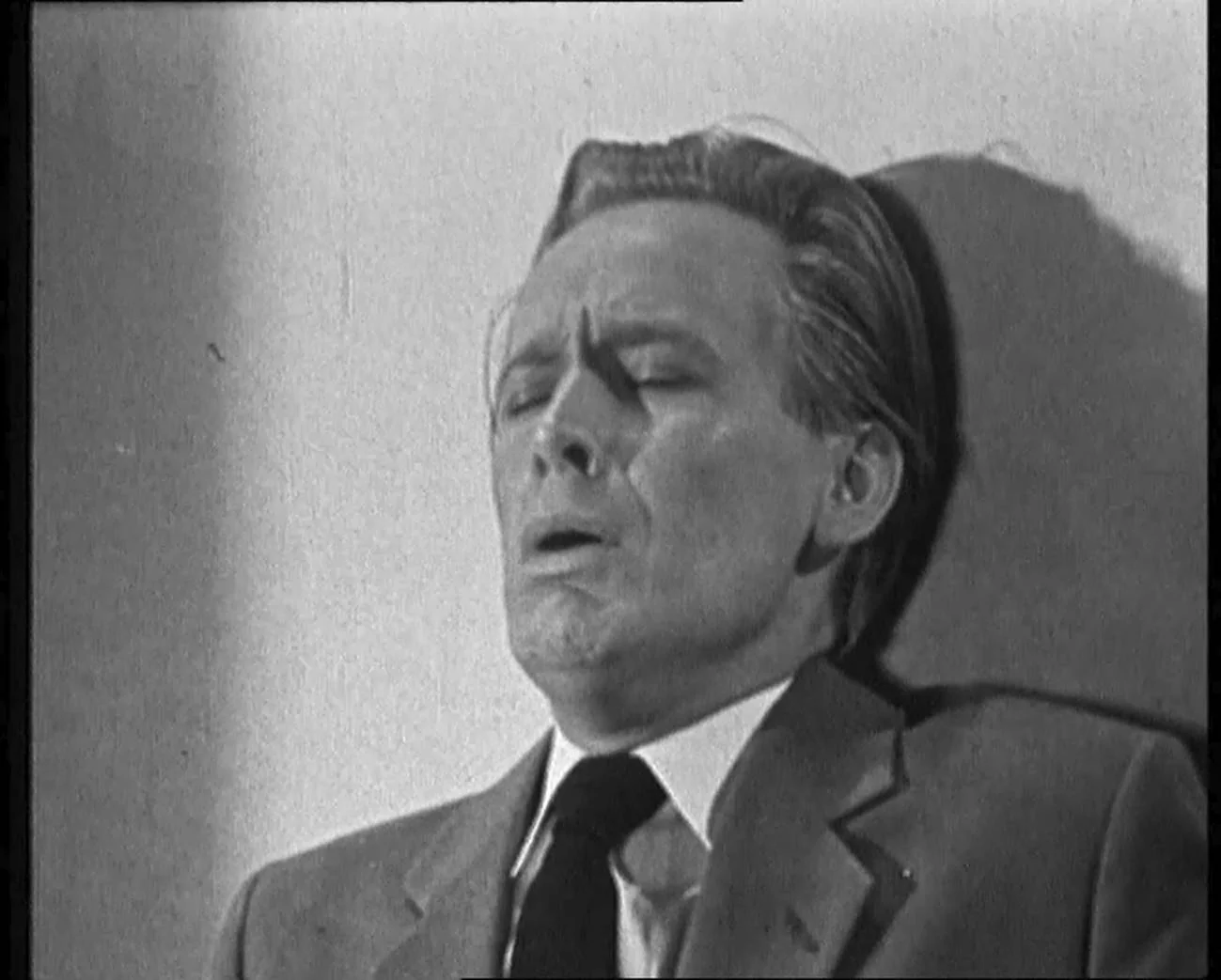
“A sense of despair, without hope” in *Det nya ansiktet*

More than the two previous films, *Det nya ansiktet* directly addresses the emotions of its spectators and attempts to make the audience identify with the role models presented in the film. To achieve this goal, the film combines a fictional plot with the more conventional documentary narrative. As the two storylines are closely interwoven, the plot is somewhat more convoluted. The fictional plot presents the story of a loving couple faced with a tragedy, which is introduced at the beginning of the film. When the young and beautiful woman drives away from her husband and home, she ends up in a car accident, leaving her with a wounded face. Over the course of the film, we learn that she has to undergo plastic surgery to restore her beauty. The camera shows very little of this, but rather focuses on the parallel story of lake regulations and their impacts on the natural beauty of the landscape. The car accident and the wounds on the young woman’s face are thus paralleled by the “wounds” in the landscape during the construction of hydraulic infrastructure. Correspondingly, plastic surgery finds its parallel in the work of landscape architects and landscaping measures aimed at creating new landscapes in the wake of lake regulations. Now, towards the middle of the film, while the beauty of both the woman and the landscape are being restored, the camera lets the spectators partake in the feelings of the young woman’s partner, who is ostensibly overwhelmed by his emotions (see Fig. 4). He is the film’s protagonist and the relatable role model, and it is his emotions that count. As the narrator comments, while identifying himself with the young man and collapsing both stories into one: “There comes a day when the landscape is one big wound and we don‘t think it can be healed, when the face that the eye has become used to has lost its beauty, and we who remember it feel a sense of despair, without hope.”

As in the two previous films, *Det nya ansiktet* places great weight here on the emotional impact of lake regulations. And again, it frames these emotions as a variant of fear and sorrow: paralyzing despair and hopelessness as well as a nostalgic longing for the past. It would not have escaped the contemporary audience of the film that the emotions displayed by the husband, seeing his wife undergoing plastic surgery in this central scene, reflect the emotions that Vattenfall imputed to nature conservationists, the target audience of the film. And it is these emotions which need to be overcome by going through a process of adaptation. While *Den nya sjön* advocated behavioral adaptation through rationalization and the building of mutual trust, *Det nya ansiktet* presents a role model for an emotional adaptation based on the reappraisal of memory.

**Fig. 5:**
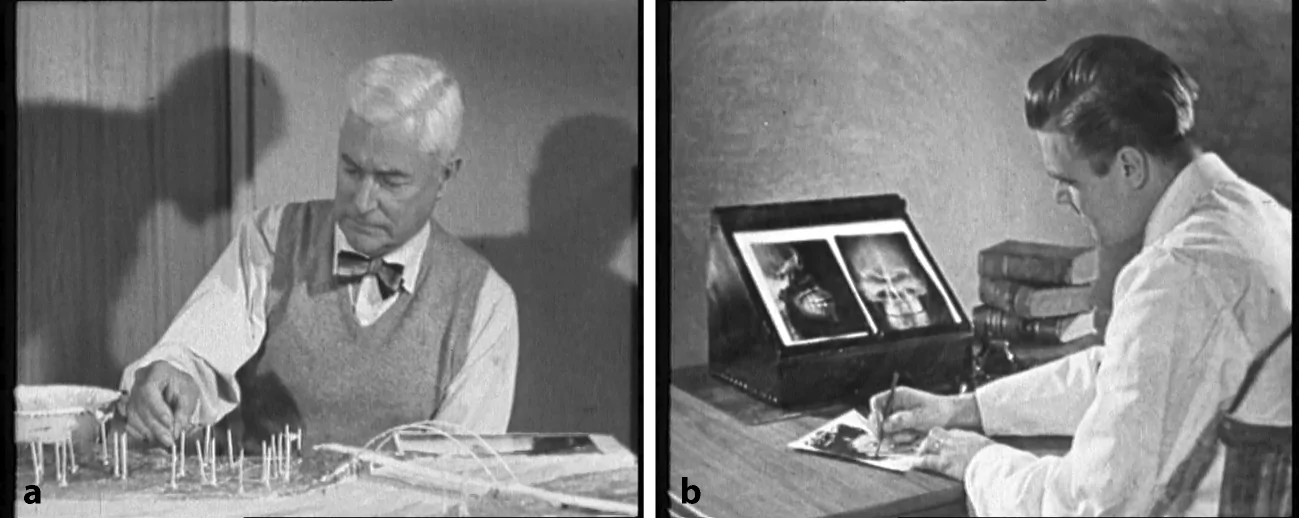
The landscape architect and the plastic surgeon in *Det nya ansiktet*

Already at the very start of the film, the narrator identifies “memory” (*minnet*) as the central problem to be resolved, as he muses: “We don’t think about, how accidental and personal our impressions are of nature’s beauty and the beauty of people. And we don’t want these impressions to change. They stay in our memory, and memory is conservative.” In order to cope with painful emotions provoked by lake regulations, this skewed memory has to be overcome. And the film presents a whole array of arguments to rationalize the emotional impact of lake regulations and to underscore Vattenfall’s role as a support for individual adaptation processes. Thus, in the exposition, the narrator argues that “[c]hanges have always hit nature and men” and that, at least in the case of naturally occurring changes, people “have come to terms with them.” He postulates furthermore that “we are in absolute need of” electricity. Thus, even if change is painful, “[w]e need to come to terms with conscious interventions, just as we do with accidental ones.” Here, *Det nya ansiktet *reasserts the fatalism of *Öden bortom horisonten*, but gives it a new twist. By comparing the impacts of lake regulations on the landscape with a car accident, it frames them as a risk inherent in modernization: “But we don’t stop driving a car because accidents happen” and, accordingly, lake regulations necessary for electricity production cannot simply be stopped because of their side effects (cf. Zimmer 2024). As in *Den nya sjön*, acceptance and adaptation are thus the only option, while Vattenfall offers professional support to those adapting: *Det nya ansiktet* highlights the work of landscape architects, who can help the landscape “get a new face that respects the characteristic features of nature.” Just like plastic surgeons, these landscape architects go about their work “with care and foresight” (see Fig. 5)—they are at least as trustworthy and reliable as the land surveyors or the local office in *Den nya sjön*. Thus, finally, the new landscape, ennobled through the work of competent landscape designers, can be beautiful in itself. Accordingly, the narrator ends his speech by demanding: “Let us abandon the one-sidedness of our memory and learn to see the positive in the new face. It is not less beautiful because it is different.” Even though the film leaves this open, the hopeful music accompanying this closing scene, in which the woman with the restored face walks up to her husband, leaves no doubt that he will heed the call for acceptance and eventually adapt to the new circumstances. Without explicitly speaking of “adaptation,” *Det nya ansiktet* nevertheless tells the story of a successful adaptation to the changes brought by the “new times.”

The three films from Vattenfall’s film corpus which I have analyzed here are remarkable because they give an insight into how Vattenfall—an Agency primarily concerned with river engineering—explored film and other PR materials as emotional engineering techniques during the 1950s. Quite contrary to its earlier PR work, Vattenfall now explicitly engaged with emotions of the public and tried to put them to strategic use in the heated conflicts over lake regulations. That Vattenfall’s press service depicted the emotions of its opponents as characterized primarily by fear, despite the much more complex emotional landscape which I sketched in the introduction, might not have been a deliberate choice, but rather a naïve obeisance to conventional tech-fear narratives. In any case, this framing facilitated a specific narrative of adaptation according to which Vattenfall generously offered help and guidance in alleviating and rationalizing fears through reliable information and transparency, as well as through the trustworthy and approachable behavior of Vattenfall’s representatives.

This “system of feeling” staged in Vattenfall’s films then had far-reaching implications for how Vattenfall publicly imagined the acceptance crisis which the Agency found itself in with the conflict over lake regulations in the 1950s: it contributed to an individualization and a depoliticization of the conflict. Vattenfall’s opponents—both affected local people and nature conservationists—consistently appear as individuals in need of guidance, rather than as organized groups and associations which posed an actual threat to Vattenfall’s hydroelectricity schemes. These included most prominently the Swedish Society for Nature Conservation (Svenska Naturskyddsföreningen), the Swedish Tourist Association (Svenska Turistföreningen), the Association for Cultural Heritage (Samfundet för Hembygdsvård), and the more monothematic Co-operative Committee for Nature and Landscape Conservation in Connection with the Use of Hydropower (Samarbetsnämnden för natur- och landskapsvård i samband med vattenkraftens utnyttjande), the latter founded as an umbrella organization in 1952; and, too, the subsequent Nature Conservation Delegation (Naturvårdsdelegationen), which drove much of the debate leading up to the “Peace of Sarek” a decade later (cf. Vedung & Brandel 2001: 60–97). Vattenfall clearly saw the work of these organizations as the central threat to its business—quite opposed to the indigenous Sami. Despite the heavy impact of Vattenfall’s hydropower schemes on their territory (cf. Öhman 2007; Össbo 2014; Össbo & Lantto 2011), and despite an incipient organized indigenous protest against lake regulations (cf. Lantto 2003), the Swedish Sami figured only very marginally in Vattenfall’s films (cf. Zimmer 2026, 2022), which testifies to the racism they faced and their accordingly low standing in public debates of the period. Even while addressing specific aspects of the controversies over hydropower exploitation, Vattenfall’s PR materials do not frame the conflict as a political one. Apart from focusing on individual adaptation stories, Vattenfall’s films repeatedly claim that the decision as to whether or not a lake is regulated, and under what conditions, is entirely and (according to *Den nya sjön*) “impartially” taken by the Water Law Courts.^14^ The expansion of hydroelectric infrastructure does not appear as a political choice made by Vattenfall; rather, Vattenfall only appears as a friendly figure who guides and supports people in their individual adaptation to the inevitable exigencies of the “new times.”

## Adaptation and the Mythology of Modern Sweden

My analysis of Vattenfall’s films up to this point has demonstrated how the Agency started engaging with emotions of the public in the context of a severe acceptance crisis and conflicts over the impacts of “lake regulations” on landscapes and people’s livelihoods. However, this context does not sufficiently explain why Vattenfall’s press service chose to frame these conflicts and the engagement with the emotions connected to them as stories of adaptation. Why did adaptation narratives have such high plausibility that Vattenfall’s PR could self-evidently call upon them? As I will argue in this section, these narratives only made sense within a wider discourse on “adaptation,” one which informed internal public relations during the 1950s and contemporary trends in industrial psychology and sociology, as well as broader political debates on modernization and the future of Sweden’s welfare state. I demonstrate on the following pages how this adaptation discourse dovetailed nicely with Vattenfall’s efforts to manage public opinion and to regain acceptance for its modernization program, thus lending Vattenfall’s adaptation narratives a particularly high plausibility.

Vattenfall’s initial engagement with matters of adaptation was in internal public relations. As I mentioned above, Vattenfall first ventured into PR in the field of internal communication, when the Agency installed a dedicated press service and started publishing the staff magazine *Vi i Vattenfall* in 1948. *Vi i Vattenfall* came into existence at the intersection of several developments in the Agency and beyond. Vattenfall had seen a dramatic growth of the workforce since World War II, which also engendered a generational shift. Thus, the declared aim of *Vi i Vattenfall* was to “help promote understanding of the Agency’s endeavors and create ever better contact between everyone at Vattenfall [*alla vattenfallare*],” as Åke Rusck, the then newly appointed director general, put it in the editorial of the first issue (Rusck 1948). Furthermore, the creation of *Vi i Vattenfall* was closely coupled with the installation of so-called “work councils” (*företagsnämnder*) at Vattenfall in January 1948. All larger companies in Sweden had been legally obliged in 1947 to set up these regular forums, in which representatives from management as well as from employees and workers would meet, “for information and consultation as well as for promoting good operating and working conditions” (Horss 1948). The debates in the work councils were a central topic, especially in the first volumes of *Vi i Vattenfall*. A programmatic article on the first pages of the first issue gave insights into “what we hope for from the work councils.” The topics named here by various council members were broad, ranging from matters of work safety and occupational health to the improvement of in-house training, the implementation of industrial democracy (*företagsdemokrati*), and the wish “to create trust between the Agency’s management and the employees” (*Vi i Vattenfall* 1948—the quote is on p. 4). The overarching aim, which almost all of the council members in the article attributed to the work councils, was the improvement of “well-being” (*trivsel*), and director general Åke Rusck had given this aim a similarly central value in the aforementioned editorial: “Well-being and work satisfaction [*Trivsel och arbetsglädje*] are values that we must safeguard” (Rusck 1948).

This focus on “well-being” at Vattenfall arose at a time, when “well-being” and “adaptation” were broadly debated in Swedish industry at large and, more specifically, in the developing fields of industrial psychology and sociology. An early study in this latter field was published in 1952 by Torgny Segerstedt, who held the first chair in sociology in Sweden (at Uppsala from 1947), and his student, Agne Lundquist. *Människan i industrisamhället* (Man in Industrial Society), as the study was called, focused on the “question of how people can orient themselves and adapt in their work, what is usually summarized under the term ‘well-being’” (Segerstedt & Lundquist 1952: 5).^15^ “Adaptation” and “well-being” were thus central objects of inquiry in Segerstedt and Lundquists’ study. Writing three decades later, sociologists Torsten Björkman and Karin Lundqvist singled out *Människan i industrisamhället* as something of a model for a whole school of thought in industrial sociology and psychology in postwar Sweden, which they call the “adaptation paradigm” (cf. Björkman & Lundqvist 1981: 34–35). This paradigm was strongly inspired by the American Human Relations movement, which sought to improve relations between people in the company, ultimately in order to increase production. Research within the Swedish “adaptation paradigm” thus sought social scientific answers to issues that Swedish industry faced after World War II. These encompassed high mobility among workers, absenteeism, and especially the political situation after the war, as it became clear that the power balance and the industrial peace which industry and trade unions had reached agreement on in Saltsjöbaden in 1938 would be lasting, making the sustained cooperation of both sides a necessity (ibid.: 30).

These studies were widely received within Swedish industry. For instance, a shorter summary of *Människan i industrisamhället* was published a few years later in a series edited by the Personnel Administrative Council (Personaladministrativa rådet, in short: PA-rådet/PA-Council) (Lundquist 1957). The PA-Council had been established in 1952 by the Swedish Employers’ Confederation (Svenska Arbetsgivareföreningen, SAF), as a reaction to the establishment of the work councils and in order to strengthen industrial psychology and sociology and the dissemination of their research findings among Swedish industry (cf. Björkman & Lundqvist 1981: 32; Björkin 2022: 81–83). Given its attachment to SAF the PA-Council mainly represented the interests of the private sector of Swedish industry, similar to the think-tank which had initiated the research for *Människan i industrisamhället*. Nevertheless, the adaptation research by Segerstedt, Lundquist, and others was also received with great attention in the public utility Vattenfall: *Vi i Vattenfall* published a review of *Människan i industrisamhället* in 1955, complementing a large number of articles on various aspects of “well-being” in the Agency (cf. Harling 1955).

In a recent study on *Post-War Industrial Media Culture in Sweden*, media scholar Mats Björkin (2022) has argued that the establishment of the work councils at the end of the 1940s drove a profound shift in industrial media use in Sweden. As the human relations and public relations literature of the period incessantly highlighted, good communication between management, employees, and workers had now become a prerequisite for economic success (cf. Segerstedt & Lundquist 1952: 36–37 and passim; Höglund 1953; Lindström 1958). According to Björkin, the shift in industrial relations manifest in the establishment of the work councils thus entailed, among other things, “a move from using industrial films as documentation or advertising … to a use of film as an information and communication technology” (Björkin 2022: 93). The organizing metaphor and ideal of good and productive communication in the industrial media discourse of the period was “contact.” According to Björkin, the ideal of “contact” represented

“a middle path to follow between individualism and the mass ideologies of the interwar years. … It was supposed to uphold personal exchange at a time when both public authorities and private companies grew larger. It was an attempt to use the old and new media technologies without losing personal interaction” (ibid.: 12).

Vattenfall’s internal and external communication very clearly adhered to this ideal of “contact,” as can be evidenced by the aforementioned Åke Rusck editorial or the press service’s shift towards using film as a central medium for its PR work. Accordingly, Vattenfall is also an important case study for Björkin. However, from the perspective I have developed here, next to the ideal of “contact” one should place the ideal of “adaptation,” which is missing in Björkin’s account. If contact was “a set of technologies of interaction” (ibid.: 12), then the goal of this interaction, the cultural and political program to be enacted through contact, was adaptation.

This adaptation discourse was not restricted to the managerial side of the Swedish economy, however, as the labor movement took just as much interest in the debates on adaptation. Thus, the Worker’s Educational Association (Arbetarnas Bildningsförbund) organized a major conference in Stockholm in 1956—under the title *Människan i dagens och morgondagens samhälle *(Man in Today’s and Tomorrow’s Society)—which aimed to offer insights into the “human consequences of the technical and economic development” (Arbetarnas Bildningsförbund 1956: 7).^16^ Most notably, Tage Erlander, the Social Democratic prime minister, also participated in the conference with a short speech on “Society, adaptation and public opinion.” In his speech, Erlander campaigned for his government’s investments in what he called “cultural policy” (*kulturpolitik*), especially concerning education and research. According to Erlander, investing in these sectors was imperative as a reaction to technological change, which he saw as something of a driver of history: “New scientific discoveries and technical inventions lead to new forms of production and society, and people are forced to adapt to the new that is evolving” (Erlander 1956a: 266). Only if politicians addressed this necessary “adaptation process” in the right way, could “our standard of living” be maintained “in the international competition”; only then could the “rootlessness, the anxiety, and the fear” (*[r]otlösheten, oron och ångesten*) in society be successfully combated and society reorganized, so “that conflicts for the individual become as small as possible” (ibid.: 267). Already the previous year, Erlander had developed similar thoughts at another conference which his party and the Swedish Trade Union Confederation (Landsorganisationen) had organized under the title *Tekniken och morgondagens samhälle* (Technology and Tomorrow’s Society). This conference had focused more optimistically on the prospects of automation and atomic energy (for instance, with Vattenfall’s director general Åke Rusck as one of the speakers); yet already here, Erlander had pointed to the political challenges posed by technical change: “Our problem will be to create in a democracy the society of cooperation and collaboration that technology and modern large-scale production require” (Erlander 1956b). In both of his speeches, Erlander thus invoked a “linear model of innovation” common to the postwar period, according to which technical change is an inevitability and society and individuals need to embrace change and adapt accordingly (cf. Godin 2006; Rahm 2021: 39–42). Furthermore, he thereby drew on ideas of social engineering which were part and parcel of the mythology of modern Sweden (cf. Ruth 1984).

As various historians have noted, ideas and practices of reordering society and regulating individual behavior in order to counter the centrifugal forces of modernization—social engineering in short—had a particularly firm footing in twentieth-century Sweden (cf. Etzemüller 2010; Hirdman 1989). Thomas Etzemüller has argued that this strong influence of social engineering ideas has to be seen in close entanglement with the corporatist structure of Swedish society. Since its origins in the period around 1900, ideals of conscientiousness (*skötsamhet*) and mutual agreement (*samförstånd*) had been rehearsed and established in the Swedish worker’s movement, revival movement, temperance movement, and the various other associations which came to be summarized as the popular movements (*fölkrörelserna*), and which had laid the cultural foundations for the Swedish welfare state. The culture of Swedish corporatism developed in these movements demanded from the individual an orderly, controlled way of life. Paired with the inclination to avoid conflict and seek compromise, it placed the collective above the individual and accordingly demanded adaptation from the individual (cf. Etzemüller 2010: 111–12; furthermore, Ambjörnsson 1988).

Already in the 1930s—the period usually identified as the heyday of social engineering in Sweden—a group of architects (and one art historian) published the notorious modernist manifesto entitled *acceptera *(Asplund et al. 1931).^17^*Acceptera* dramatically juxtaposed a vision of “A-Europe”—the heavily industrialized, high-tech, networked, urban Europe—with that of “B-Europe”—the traditional, agrarian, rural, isolated Europe. To the authors of the manifesto, it was evident and inevitable which path Sweden had to choose and the imperative title of the book (“accept”!) clearly announced the attitude which the Swedes should adopt towards modernization. On a superficial level, *acceptera* was concerned with architecture and design, but it ultimately was an expression of how Swedish modernists tried to “tackle … issues that essentially belonged on the level of social organization as a whole” (Mattson & Wallenstein 2007: 64). It had the “primary task … to make the individual identify with the project of modernization,” as Helena Mattson and Sven-Olov Wallenstein have argued (ibid.: 62).

In sum, the adaptation discourse of the 1950s needs to be seen in this tradition of social engineering discourses. It is no coincidence that twenty years after *acceptera*, Segerstedt and Lundquists’ *Människan i industrisamhället* follows the same—albeit more nuanced—narrative of modernization, highlighting that Sweden had experienced a profound shift from an agrarian to an industrial society within just one generation. This profound shift evidently produced a number of “adaptation problems”: “We have assumed that for many, the substitution of the rural working environment for industry was surrounded by strong emotions. … These feelings may still influence people’s attitudes and their willingness to accept industrialized society” (Segerstedt & Lundquist 1952: 26–27). In essence, this is also the narrative presented in Vattenfall’s PR of the 1950s: “The new times” bring the transition from an agrarian, isolated lifestyle to an industrialized and networked one. However, strong emotions, which Vattenfall’s PR interpreted as fear, inhibited acceptance of modernization and therefore needed to be addressed as “adaptation problems.”

For Vattenfall’s press service then, it might have seemed self-evident to follow the contemporary adaptation discourse when trying to mitigate the Agency’s acceptance crisis of the 1950s. As the press service gradually shifted its focus from internal relations (in response to the creation of the work councils) to external relations (in response to the conflicts over lake regulations) throughout the 1950s, it seems that the adaptation narratives first developed in the context of internal and human relations were simply translated from one domain to the other. Likewise, the practices of “contact” developed in the engagement with the work councils were applied to Vattenfall’s external public relations activities during the 1950s. While Vattenfall’s films were regularly shown in the pre-program of cinemas, the press service made a major effort to organize interactive film screenings for the Agency’s workforce all over the country as well as for “specially invited guests,” which referred to representatives not only from the government and various branches of Swedish industry, but also from nature conservation organizations—in short “people with whom we come into *contact* in one context or another in our work.”^18^ Furthermore, during the 1950s, Vattenfall also produced a series of salvage films, documenting specific landscapes and the livelihoods of their inhabitants before they were permanently altered by lake regulations (cf. Zimmer 2026). These films were dedicated to the portrayed communities and handed over in copy as gifts to representatives of the respective municipality as a sign of good “contact” (cf. *Vi i Vattenfall* 1956a).

Ultimately, the discourse of adaptation, firmly established as it was in the sociotechnical and wider political imaginaries of twentieth-century Sweden, shaping much of the wider industrial discourse as well as ideas on modernization in the political sphere, came with an immense plausibility and with specific rhetorical advantages. According to Segerstedt and Lundquist, “adaptation” could be defined as a measure of the match between (“subjective”) expectations of a situation and the actual (“objective”) situation, for instance the specific working conditions in a certain company. Successful “adaptation” could thus imply an adaptation of both the individual’s expectations as well as of the specific conditions in question. However, as Björkman and Lundqvist have argued, the focus on adaptation and well-being in the postwar industrial psychology and sociology of the “adaptation paradigm” had a strong tendency to individualize work-related issues, while the actual influence of the work councils often remained very limited and became a “communication channel” for management rather than an actual instance of participation or industrial democracy (Björkman & Lundqvist 1981: 33; cf. Isacson 2019: 313). Similarly, as I have shown here, the political discourse on adaptation asserted that technology inevitably and inescapably drove history, making societal and individual adaptation to technological change seem less a matter of political choice than a sheer necessity. When Vattenfall chose to frame conflicts about lake regulations as matters of adaptation, it could conveniently capitalize on these very same aspects of the adaptation discourse. Here, the focus on adaptation likewise helped to depoliticize and to individualize the conflict over technological choice.

## Adapting to Modernity—Conclusion

In this paper, I have argued that Vattenfall’s PR work started addressing the emotions of the public in response to a major acceptance crisis. During the 1950s, conflicts around so-called lake regulations and their consequences, such as resettlement or impacts on nature conservation, gained a strong momentum, eventually leading to the “Peace of Sarek” in 1962, which was meant to establish a lasting compromise between the conflicting parties. However, that Vattenfall specifically chose narratives of adaptation for framing these conflicts in its PR work can only be explained by examining the wider discursive and cultural contexts beyond the immediate conflicts at hand. In joining the broad contemporary discourse on “adaptation” in Swedish industry, politics, and society, Vattenfall’s PR could call upon the social engineering ideals of the Swedish welfare state, thereby individualizing and depoliticizing the conflict.

As I have shown, Vattenfall’s PR department used films as an emotional engineering technique. Producing and distributing films did much more for the Agency than documenting or depicting certain emotions. Rather, Vattenfall’s films actively ascribed specific sets of feelings to specific groups, offering role models with the aim of actively shaping the emotions and behaviors of their audiences. Despite the broad range of emotions Vattenfall encountered in its contacts with various groups publicly opposing “lake regulations,” and despite the broad range of emotions experienced within the company in these conflicts, Vattenfall’s films and other PR work projected a specific “system of feeling” (Rosenwein 2002), both convenient and conventional, in which the opponents to hydropower were characterized as fearful, while Vattenfall appeared as a rational and friendly helping hand. The case of the Swedish adaptation discourse thus gives an insight into the fabrication of a conventional standard narrative of “technology acceptance” and the attribution of specific emotions attached to this narrative. My case study therefore highlights the emotional attentiveness required from historians of technology in their analysis of debates on “technology acceptance.” If we want to move away from clichés of “anxious enemies of technology” on the one hand and rational proponents of technology on the other, and if we want to deconstruct top-down narratives of “technology acceptance,” we need to pay close attention to who expresses which emotions and who ascribes what emotions to whom in conflicts over technological choice.

Even if what I have presented here, is perhaps a *typically* Swedish story, it should not be seen as an *exclusively* Swedish story. In other regions, hydropower companies rarely relied on explicit adaptation narratives in their PR.^19^ Nevertheless, narratives of adaptation can be found in industrial discourses in other regions too—one need only think of the US Human Relations movement or the West German initiatives for the “Humanization of Work” (cf. Kleinöder et al. 2019; Kellershohn 2021). They also pervade the PR and industrial film literature of the mid-twentieth century in other regions. For instance, Friedrich Mörtzsch, head of AEG’s PR department in the 1950s, wrote in an industrial film handbook that the use of industrial films in the US had proven its potential to “facilitate the adaptation of man to his engineered environment” (Mörtzsch 1959: 26). Even beyond the more confined industrial discourses, “adaptation” became a central concept in physiological theories of stress (cf. Haller et al. 2014) or in ecology, biology, and the life sciences in the postwar period, most notably in the “Human Adaptability” arm of the International Biological Programme (IBP) running from 1964 to 1974. This is more than a coincidence. The focus on “adaptation” in the IBP arose “[d]uring the late 1950s and early 1960s, [when] there was a heightened concern with anthropogenic effects on the environment that coincided with the postwar rise in science and technology,” as Michael Little has argued (2012: S132). Just like the industrial adaptation discourse, the biological adaptation discourse can thus be interpreted as a mode of responding to the challenges of rapid modernization. These discourses then promise to offer a rich transnational and transdisciplinary field in which the relation of humans to technological change was negotiated in the second half of the twentieth century.

## Footnotes

1 All translations by the author.

2 In an internal document Cederholm, Charlie. *Vattenfalls externa PR-verksamhet. Bakgrund, idéer, handlingsprogram. *1970, p. 12, Vattenfalls Kulturarvskommitté, Solna 3888.

3 On Vattenfall’s earlier history, see Kaijser (1995) and Jakobsson (1995) among the English literature. On the postwar conflicts, see the English overview in Bernes & Lundgren (2009: 134–43) and the more focused study by Sundin (2004). For more detailed accounts of the postwar period, see the Swedish literature, especially Vedung & Brandel (2001); Anshelm (1992); Forsgren (1993).

4 I confess to taking for granted some of these narratives in my earlier readings of Vattenfall’s PR work (Zimmer 2022, 2024). In this paper I build on findings from these earlier publications but expand and revise some of my interpretations.

5 I give a more detailed discussion of the press service and its history in Zimmer (2026).

6 All quotes from: Cederholm, Charlie. *Filmproduktion*. 16.03.1959, p. 1, Riksarkivet, Arninge (RA) 420579/38/F1AA/2204.

7 Cf. Förteckning över Vattenfallsstyrelsens filmer 1946–1959 och dess medarbetare. 03.06.1959, RA 420579/14/F7/1; CP visning av film under 1959. 22.03.1960, RA 420579/14/F7/1.

8 Besides the ones discussed here, these are *Suorva, Järkvissle, Långbjörn, Kultsjöån, Tärna, Den starka älven*, and *Stora Lule älv*. As opposed to the films analyzed in this paper, these are more site-specific and less general in their claims, and would therefore only add more detail to the analysis. I discuss them from a different angle in Zimmer (2026).

9 Cf. *Vattenfalls filmer på biograf 1955–59.* 03.06.1959, RA 420579/14/F7/1; Cederholm, Charlie. *Letter to Åke Rusck. Filmen om sjöregleringarna.* 13.12.1955, RA 420579/38/F1AA/2204. *Öden bortom horisonten*: Sweden 1956; commissioner: Vattenfall; production: Ivan Christoferson, Svensk Filmindustri; direction & script: Gösta Werner; camera: Sten Dahlgren; narrator: Åke Engerstedt; 35 mm, color, sound, 14 min. URL: https://www.filmarkivet.se/movies/oden-bortom-horisonten/ (14.04.2023). On Gösta Werner (with but a brief mention of this film), see Stjernholm (2018).

10 Cederholm, Charlie. Letter to Jonas Norrby. En allmänn good-will-film om sjöregleringar. 20.04.1955, RA 420579/38/F1AA/2205.

11 Cf. *Vattenfalls filmer på biograf 1955–59.* 03.06.1959. RA 420579/14/F7/1; *Vi i Vattenfall *(1957). *Den nya sjön*: Sweden 1957; commissioner: Vattenfall; production & direction: Ivan Christoferson, Europafilm; script: Charlie Cederholm; camera: Ingvar Borild; color, sound, 13 min. URL: https://www.youtube.com/watch?v=YyolhXSKZd4 (23.08.2023).

12 Cf. Cederholm, Charlie. *Meddelande “Film om naturskydd”. *09.04.1958, RA 420579/38/F1AA/2204; Cederholm, Charlie. *Förteckning över färdigställda och planerade filmer.* 28.09.1956, RA 420579/38/F1AA/2205; *Vattenfalls filmer på biograf 1955–59.* 03.06.1959, RA 420579/14/F7/1. *Det nya ansiktet*: Sweden 1958; commissioner: Vattenfall; production: Ivan Christoferson, Charlie Cederholm, Kinocentralen AB; direction & script: Alex. Jute; camera: Karl-Erik Alberts; narrator: Lars Ekborg; cast: Jan Malmsjö; color, sound, 12 min. URL: https://www.youtube.com/watch?v=eMayNCnycpY (23.06.2022). *Det nya ansiktet *has had something of a reputation among Swedish historians for a while, and it has been discussed from various angles in Krohn Andersson (2012); Björkin (2022); Zimmer (2022, 2024).

13 Cf. Cederholm, Charlie. *Förteckning över färdigställda och planerade filmer.* 28.09.1956, RA 420579/38/F1AA/2205.

14 In fact, it was not until 1956 that Swedish Water Law Courts, which were traditionally very much in favor of hydropower exploitation, first ruled against a lake regulation out of consideration for the interests of nature conservation and the local population, cf. Vedung & Brandel (2001: 69 f.).

15 The quote is from the preface written by Ragnar Sachs, executive director at Nordiska Kompaniet (NK), and Ture Flyboo, trade unionist in the Swedish Trade Union Confederation. A volume two was published a few years later as Segerstedt & Lundquist (1955).

16 I thank Lina Rahm for pointing me to the adaptation debate in the labor movement and for providing me with a copy of *Människan i dagens och morgondagens samhälle*.

17 An English translation is available in Creagh et al. (2008).

18 Cederholm, Charlie. *Visning i Stockholm av filmprogrammet för speciellt inbjudna.* 09.12.1955, RA 420579/38/F1AA/2204 (my emphasis). Cf. Cederholm, Charlie. *Filmförevisningar i Stockholm för särskilt inbjudna.* 12.10.1956, RA 420579/38/F1AA/2005.

19 See my comparative study Zimmer (2022). The case studies from France and Germany treated here (besides Vattenfall) can also be read from a perspective on adaptation, but there is no explicit discourse on adaptation, or only much later. Thus, an EDF-commissioned film from 1978, *Le Verdon, de l’eau pour les hommes *(https://vimeo.com/226440658), frames resettlement as an “adaptation process,” much the same way as Vattenfall’s films had done twenty years earlier.
